# Are ticks venomous animals?

**DOI:** 10.1186/1742-9994-11-47

**Published:** 2014-07-01

**Authors:** Alejandro Cabezas-Cruz, James J Valdés

**Affiliations:** 1 Center for Infection and Immunity of Lille (CIIL), INSERM U1019 – CNRS UMR 8204, Université Lille Nord de France, Institut Pasteur de Lille, Lille, France; 2 SaBio. Instituto de Investigación de Recursos Cinegéticos, IREC-CSIC-UCLM-JCCM, Ciudad Real 13005, Spain; 3 Institute of Parasitology, Biology Centre of the Academy of Sciences of the Czech Republic, České Budějovice, 37005, Czech Republic

**Keywords:** Ticks, Venom, Secreted proteins, Toxicoses, Pathogens, Convergence

## Abstract

**Introduction:**

As an ecological adaptation venoms have evolved independently in several species of Metazoa. As haematophagous arthropods ticks are mainly considered as ectoparasites due to directly feeding on the skin of animal hosts. Ticks are of major importance since they serve as vectors for several diseases affecting humans and livestock animals. Ticks are rarely considered as venomous animals despite that tick saliva contains several protein families present in venomous taxa and that many Ixodida genera can induce paralysis and other types of toxicoses. Tick saliva was previously proposed as a special kind of venom since tick venom is used for blood feeding that counteracts host defense mechanisms. As a result, the present study provides evidence to reconsider the venomous properties of tick saliva.

**Results:**

Based on our extensive literature mining and *in silico* research, we demonstrate that ticks share several similarities with other venomous taxa. Many tick salivary protein families and their previously described functions are homologous to proteins found in scorpion, spider, snake, platypus and bee venoms. This infers that there is a structural and functional convergence between several molecular components in tick saliva and the venoms from other recognized venomous taxa. We also highlight the fact that the immune response against tick saliva and venoms (from recognized venomous taxa) are both dominated by an allergic immunity background. Furthermore, by comparing the major molecular components of human saliva, as an example of a non-venomous animal, with that of ticks we find evidence that ticks resemble more venomous than non-venomous animals. Finally, we introduce our considerations regarding the evolution of venoms in Arachnida.

**Conclusions:**

Taking into account the composition of tick saliva, the venomous functions that ticks have while interacting with their hosts, and the distinguishable differences between human (non-venomous) and tick salivary proteins, we consider that ticks should be referred to as venomous ectoparasites.

## Introduction

As haematophagous (blood sucking) arthropods, ticks are mainly considered as ectoparasites that use their salivary constituents to successfully obtain a blood meal by targeting major physiological pathways involved in host defense mechanisms [1]. Ticks constitute an important pest affecting agricultural development, as well as domestic animal and human health since they transmit a variety of infectious agents. Tick saliva has been described as a complex mixture of pharmacologically active compounds with implications for pathogen transmission [1]. From a functional and evolutionary point of view, Fry and colleagues [2], considered the feeding secretions of some haematophagous invertebrates (such as ticks) as a specialized subtype of venom. Certainly, Ixodida, that includes hard and soft tick species, is proven to be a venomous taxonomic Order in Chelicerata [3]. In fact, the bite from a single tick can produce several types of toxicoses [4]; paralysis being the most common and recognized form of tick-induced toxicoses [3,5].

Tick paralysis is an ascending motor paralysis produced by an impairment of neurotransmission, possibly due to the blockade of ion channels involved in the depolarization of nervous tissue [6]. This form of polyneuropathy is mainly associated with the acquisition of a blood meal by female ticks and will spread to the upper limbs of the host, causing incoordination and, in some cases, ending with respiratory failure and death [4]. Nevertheless, evident signs of toxicoses (e.g., paralysis) are not a *sine qua non* effect from the tick bite as in the case of other venomous taxa, such as snakes, spiders, scorpions or pseudoscorpions. This observational scarcity is perhaps the reason ticks are not considered venomous animals. Thus, tick saliva as venom has rarely been mentioned in parasitological literature, with the exception of a few examples (e.g., as in [7]).

Traditionally, venom was defined as a toxic fluid that inflicts an abrupt death or paralysis in the host and/or prey. This archaic concept, however, partially highlights the deleterious effects of venom on the host/prey and lacks ecological relevance. After investigating many venomous animals, Fry and colleagues [2,8] extended this limited definition of venom *“as secretions produced in specialized glands and delivered through a wound (regardless of the wound size), that interferes with normal physiological processes to facilitate feeding or defense by the animal that produces the venom”*. By interfering with normal host physiological processes infers that all toxins are venomous, but not all venomous proteins are toxic. This new paradigm allows us to consider a wider spectrum of envenomation produced by a myriad of macromolecules. In our study we hypothesize that due to their salivary composition ticks are venomous animals within the phylum Chelicerata. We base our hypothesis on the following points: (i) the various toxic effects induced by ticks (ii) the convergent protein families present in spiders, scorpions and ticks; (iii) the immunomodulatory properties found in ticks saliva is also found in other venomous taxa (iv) the pattern of immune response against toxins by the host/prey is similar in both ticks and other venomous taxa; (v) the structural similarities in members of major protein families between known venomous taxa and ticks; (vi) the bimodal structural dichotomy between human (non-venomous) and tick saliva; and, finally (vii), the phylogenetic position of parasitiformes (Ixodida, Holothyrida and Mesostigmata) as a sister clade of pseudoscorpiones based on [9].

## Results and discussion

### Toxicoses phenomena within ixodida

**Figure 1:**
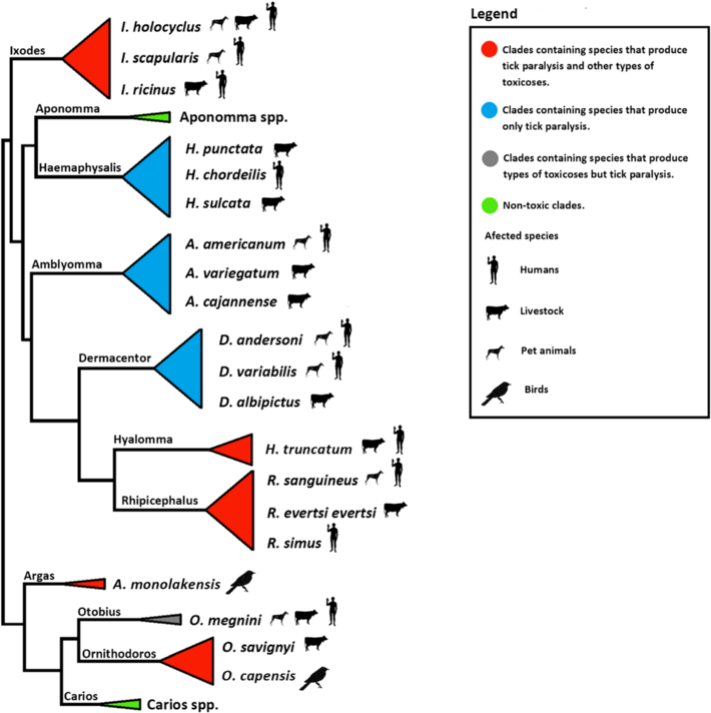
**Phylogenetic distribution of the major tick toxicoses-inducing genera.** The phylogenetic tree was compiled from published sources [18,19]. Data regarding tick toxicoses among Ixodida genera and presented tick species was collected from [3].

The Australian *Ixodes holocyclus* is perhaps the best example of a tick that induces paralysis on livestock [10], pet animals [11], and humans [12]. Tick-induced paralysis, however, is not limited to this tick species but has been reported for ~8% of all tick species from major tick genera, except Carios and Aponomma [3] (69 out of approximately 869 tick species; 55 hard tick species and 14 soft tick species). Some of these paralyses inducing tick species represented in Figure 1 are also endemic to and abundant in several geographic regions [4]. Examples in the distribution of such ticks species are the North American *Ixodes scapularis*, *Dermacentor variabilis* and *Amblyomma americanum*[13,14], the South American *Amblyomma cajannense*[15], the European *Ixodes ricinus*[16], and the globally distributed *Rhipicephalus sanguineus*[17].

Additionally, several lethal and paralysis inducing toxins have been identified in ticks. For example, the 15.4 kDa acidic salivary toxin secreted by *Ornithodoros savignyi* is highly abundant and its purified form kills a mouse within 90 minutes at a concentration of 400 μg/10 g of mouse weight [20]. Another purified basic toxin from the same tick species was shown to kill a 20 g mouse within 30 minutes after administration of 34 μg of the toxin [21]. Verified via Western blot, a 20 kDa trimeric neurotoxin was identified in the salivary glands of *Rhipicephalus evertsi evertsi* that paralyzed muscle contractions in an *in vitro* assay [22,23]. Maritz and colleagues (2001) identified a 60 kDa toxin in *Argas walkerae* that reduces [3H]glycine release from crude rat brain synaptosomes, indicating a paralytic effect. Other toxins have also been identified in tick egg extract from *Amblyomma hebraeum*, *R. e. evertsi, R. microplus*, *R. decoloratus* and *Hyalomma truncatum*, (revised in [4]). The presence of these toxins in tick eggs may be related to the protection of the egg mass against predation in natural environments – adding a new function for venoms in ticks, i.e., defense.

Besides tick paralysis, other types of toxicoses can be induced by a particular tick species, including sand tampan toxicoses by *O. savignyi*, sweating sickness, Mhlosinga, Magudu, and necrotic stomatitis nephrosis syndrome by *H. truncatum*, spring lamp paralysis in South Africa by *R. e. evertsi*, and, finally, specific toxicoses induced by *R. microplus*, *D. marginatus*, *R. appendiculatus*, *I. rekicorzevi* and *O. gurneyi* (revised in [3]). Toxicoses by *R. microplus*, *H. truncatum* and *R. appendiculatus* induce an anorexigenic effect [3], as induced by the secreted toxin Bv8 from the skin of the fire-bellied toad, *Bombina variegata*[24]. Symptoms of general toxicoses were also reported after soft tick bites that include pain, blisters, local irritation, oedema, fever, pruritus, inflammation and systemic disturbances [25]. Recently, human and canine toxicoses induced by the argasid tick *O. brasiliensis*, known as “mouro” tick, were reported and the most frequent symptoms of toxicoses induced by this tick species were local pruritus, slow healing lesions, local edema and erythema, and local skin rash [26]. Different types of immune reactions can also be included in the general scope of tick toxicoses [3,27]. Immediate and delayed skin hypersensitivity was reported in cattle exposed to *R. microplus* and *R. decoloratus* antigens [28,29], and in dogs exposed to *A. cajennense* antigens [30].

There are important factors in considering the severity of tick-induced toxicoses. (i) As stated by Paracelsus, *the dose makes the poison*. For example, *I. rubicundus* induces Karoo paralysis in South African livestock only when critical infestation densities are reached during repletion [31]. (ii) The anatomical location where the tick saliva is inoculated also seems to play a role in the toxic output. Although the tick species was not identified, a case report described a 3 year-old Indian boy with an acute onset of left-sided facial palsy secondary to tick infestation in the left ear [32]. Therefore, the proximity to a nerve (in this case the facial nerve) was important for the clinical toxic output (left-sided facial palsy). A similar case was also reported in a 3 year-old Turkish girl [33]. (iii) The duration of tick feeding is also an important factor of induced toxicoses [4]. Venzal and colleagues [34] showed that, after 3 days, laboratory mice infested with *Ornithodoros aff. puertoricensis* had initial signs of hyperaemia, followed by respiratory symptoms on day 4, and finally after 4 days the mice displayed nervous incoordination. A final factor (iv) to consider is the presence of common antigens between tick saliva and hosts. Recent episodes of human anaphylaxis after allergic sensitizations induced by bites of *A. americanum* have been reported. Patients with a history of *A. americanum* bites produced increased levels of pro-allergenic immunoglobulin E (IgE). The increased anti-tick IgE levels in these patients were correlated to anaphylactic reactions to one anti-cancer monoclonal antibody (Cetuximab) and red meats [35]. Anaphylaxis induced by *A. americanum* is provoked by the presence of specific IgE to the carbohydrate galactose-alpha-1,3-galactose (alpha-gal) that is also present in Cetuximab and red meat [35]. Interestingly, alpha-gal was recently found in the gut of *I. ricinus*, a tick that also induces anaphylaxis [36].

### The unified view of venom immune modulation and anti-venom immune responses

The haematotoxic and neurotoxic effects associated with venom exposure are widely recognized (revised in [2]). Nevertheless, all venomous animals are also constantly challenged by the host/prey or predator immune response. Studies have shown that the immune response of laboratory animals successfully counteracted venomous toxins [37,38]. In fact, natural resistance to snake venom was reported in both prey [39,40] and predator [41]. Thus, the immune system of the host/prey must constitute an important target of venoms in order to be effective. In fact, manipulating host defense mechanisms by venoms has been reported for some venomous animals like the parasitoid wasp, *Nasonia vitripennis*[42]. *N. vitripennis*, like ticks, are considered to be an ectoparasite since the *Nasonia* larvae feed on their hosts (invertebrates) without entering the host body [42]. The venom of *N. vitripennis* must suppress the immune response of the hosts in such a way that the host “allows” the parasitoid infection while simultaneously, the host will be able to control infections by other microorganism that otherwise would compete with the *N. vitripennis* larvae development [43]. Two major host defense cascades were suppressed by *N. vitripennis* venom: the phenoloxidase cascade and the coagulation cascade [43]. Several components of *N. vitripennis* venom have been suggested to modulate the host immune system, e.g., serine protease inhibitors, serine proteases, cystein-rich/Kunitz venom proteins and cysteine-rich/trypsin inhibitor-like venom proteins [43].

Manipulation of the host/prey immune system is not restricted to venomous Hymenoptera, e.g., *N. vitripennis*; for example, the haematophagous bat *Desmodus rotundus*, a venomous animal based on its salivary composition and feeding behavior [44], possesses two members of TNF-α-stimulated gene 6 (TSG-6) family that are highly expressed in its salivary glands. The TSG-6 family members have specific anti-inflammatory properties, such as the inhibition of neutrophil migration to interact with macrophage CD44 and modulation of NF-κB signaling [45]. This suggests that TSG-6 may play a feeding-facilitating role by suppressing the immune system. One well-studied example is the immune modulation induced by ticks in their hosts. The immune system manipulation by ticks is a complex process that has been recently revised [1].

Ticks are unique among hematophagous arthropods since they attach to host skin and feed for several days, while other blood-feeding arthropods (e.g. Triatomes or mosquitoes) feed little and often. Therefore, ticks need to counteract both the immediate innate immunity and the slower-developing adaptive immune responses in their vertebrate hosts. One first line of defense will be to counteract pain and itching responses of the host by targeting, for example, histamine, an immune-related mediator of pain and itch (revised in [1]). A few histamine-binding lipocalins was reported in the hard tick, *R. appendiculatus*[46]. In this regard, tick venom differs from canonical venoms since most venomous animals (e.g., wasps, bees, snakes, scorpions, spiders and jellyfish) will induce pain or an itch response. These venomous animals use their venom systems as a defensive or predatory function [47] with the desired effects of pain or itch to produce a deterrent effect. In contrast, similar to ticks, venomous haematophagous animals, like *D. rotundus* or triatomes bugs, should counteract prey/host awareness in order to feed until repletion.

After the skin is injured by a tick bite, the inflammatory response of the host will be activated. Ticks require a molecular arsenal to suppress both the cellular and molecular components of the host defenses. Tick salivary extract have been shown to reduce endothelial cell expression of the adhesion molecules ICAM-I and VCAM-I (*Dermacentor andersoni*) and P-selectin (*I. scapularis*). Reduction in adhesion molecules will reduce the extravasation of leukocytes at the site of tick attachment. The alternative pathway of complement activation is also one of the targets of the immunomodulation induced by tick saliva and thus complement inhibition activity has been reported in saliva of *D. andersoni*, *I. scapularis*, *I. ricinus*, *I. hexagonus*, *I. uriae* and *O. moubata* (revised in [1]). In addition, as a general trend, the saliva from haematophagous arthropods, including ticks, inhibit the proliferation of naïve T cell and the production of Th1 citokines [48]. One interesting example of modulating the adaptative immune response by hard ticks is Japanin. Japanin is a lipocalin that specifically reprograms human dendritic cells by hijacking the normal maturation process, even in the presence of “danger” signals like bacterial lipopolysaccharide [49]. Interestingly, Japanin promotes secretion of the anti-inflammatory cytokine IL-10 and increases expression of programmed death-ligand 1 (PD-L1), and both are involved in suppressing T cell immunity and induction of tolerogenic responses [49]. Such degree of molecular specialization has neither been described in other haematophagous arthropods nor in other venomous taxa. However, despite the immune suppression induced by tick saliva, some tick-host interactions result in immune-mediated acquired resistance to ticks after subsequent tick challenge.

Given the dynamics between induction of immune suppression by venoms and host/prey resistance development, an arms race between the host immune system and venomous components has been proposed [40]. The balance of this arms race will result in a susceptible or resistant host, prey or predators. In our revision of the topic, we found a convergence in the type of immune response that mammals display against both venom and tick saliva. Type 2 immune responses are mediated by lymphocytes T helper type 2 (Th2), IgE and IgG1 antibodies, but also by eosinophils, mast cells, basophils and, alternatively, by activated macrophages. This Th2 immune response encompasses a wider concept, namely allergies [50]. In mammals, venoms can induce allergic sensitization and development of specific IgE [37,38,51]; tick feeding also induces a Th2 polarization [1], specific IgE [52,53], and causes allergic sensitization [35]. The complex association between allergen IgE recognition with histamine secretion by mast cells and basophils that subsequently provoke uncomfortable reactions in animals has been highlighted [50]. This association goes beyond a specific neutralizing IgE antibodies response to a more complex detection of sensory stimuli by the olfactory, gustatory and visual systems that, surprisingly, may eventually result in developing aversive behaviors to specific locations or foods [50]. This suggests that the evolution of a differentiated pattern of immunity against venoms, including tick saliva, may have yet unexplored ecological implications. Another example of immune response convergence against venoms is that mast cells can be activated by the venom of scorpions without the concomitant presence of specific IgE [54], suggesting that the protective activities of mast cells is independent to the high affinity binding of IgE to the IgE receptor (FcϵRI) present on mast cells. Wada and colleagues [55] recently showed that the protective role of mast cells in resistant mice to the tick *Haemaphysalis longicornis* was also independent of FcϵRI. The above referenced studies show that (i) immune modulation may be a major function of venoms and (ii) the type of immune response elicited against the venom of ticks and other venomous taxa undergo similar immune pathways, thus tick saliva may possess venom-like molecules.

### Tick saliva; or, the structural convergence of venomous proteins with venomous functions

The types of toxicoses induced by tick bites (ranging from lethal paralysis to local hypersensitivity) is not limited to the presence of lethal toxins but also to the presence of specific tick salivary protein families common among other venomous taxa. Recent advances in sequencing technologies have revealed an amazing body of information from the salivary glands of both hard and soft ticks [56-64]. From these high-throughput investigations, several protein families have been identified that are involved in tick-host interactions. Such protein families are found in the venoms of several other Metazoan species [2]. Examples of such venomous protein families found in tick saliva are defensins [65], lectins [66], cystatins [67], lipocalins [21,68-71], hyaluronidase [72], phospholipase A2 [73], Kunitz-like peptides [56,74,75], metalloproteases [76], AVIT [77], CAP proteins (Cysteine-Rich Secretion Proteins, Antigen 5, and Pathogenesis-Related) [2] and sphingomyelinase D [2].

Not only are these protein families present in tick saliva, but they also possess major functions described in conventional venomous systems. These functions include inhibition of thrombin, fXa, fVII/tissue factor system, platelet aggregation (i.e., collagen-induced, ADP-induced), act as a GPIIb/IIIa receptor antagonist, or affect fibrino(geno)lytic activity (revised in [2]). At the molecular level, venomous agents display common characteristics, despite their numerous biochemical activities and sequence variability, such as (i) possessing a signal peptide, (ii) displaying functional versatility within a protein family, (iii) targeting short-term physiological processes and, (iv) stabilizing their tertiary structures via disulfide bonds. Finally, after being recruited as a functionally stable venomous agent, (v) duplication events occur to reinforce its adaptation (for a thorough description of said characteristics see [2]). An exception to this last property (gene duplication) is seen in platypus venom [78]. In the following sections we show the structural convergence between tick Kunitz peptides, cystatins, defensins, lipocalins, lectins and phospholipase A_2_ and their conventional venomous counterparts.

### Kunitz peptides

**Figure 2:**
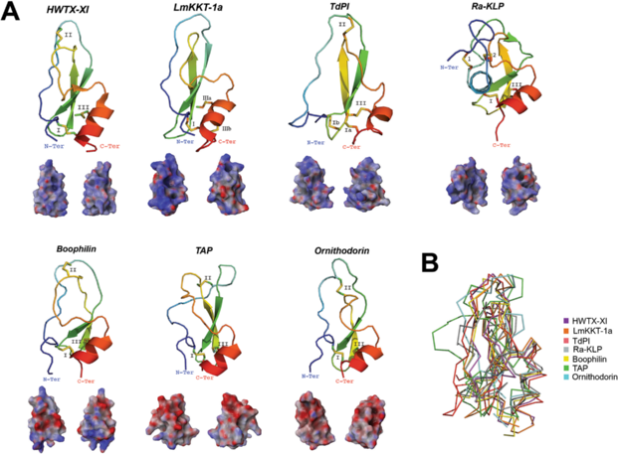
**Tertiary structures of tick salivary Kunitz peptides.** Panel **A** displays tertiary structure of toxins from spider (HWTX-XI; PDB: 2JOT) and scorpion (LmKKT-1a; PDB: 2 M01), and five tick salivary Kunitz-like peptides (PDBs: TAP-1D0D; ornithodorin-1TOC; boophilin-2ODY; TdPI-2UUX; Ra-KLP-2W8X). The tertiary structures depict the conserved disulfide bridges (indicated by roman numerals), loops, β-sheets that forms the β-hairpin, and α-helices. All structures are colored from the N-terminus (blue) to the C-terminus (red). Below each tertiary structure is the respective electrostatic potential in 180° turns (blue = positive; red = negative; white = neutral). A tertiary structural alignment in Panel **B** depicts the Cα protein backbone (color codes for each structure is presented on the right). (Note: For Panel A we used the C-terminus domain for both ornithodorin and boophilin since these possess two Kunitz-domains).

Kunitz peptides were named after Moses Kunitz who first discovered it in 1936 from bovine pancreas [79]. Since then, expression of the Kunitz protein family has been found in basically all kingdoms of life. Recent reports show that Kunitz peptides have undergone a massive gene expansion by gene duplication in the salivary glands of both *I. scapularis*[56] and *I. ricinus*[80], possibly due to specific selective pressures during the evolution of the tick-host interaction [81]. The Kunitz structure has been described with diverse functions in several venomous animals, including spiders and scorpions. Some Kunitz peptides from venomous animals possess dual activities by inhibiting both proteases and ion channels; examples of such toxins are LmKKT-1a from the scorpion *Lychas mucronatus*[82] and Huwentoxin-XI (HWTX-XI) from the spider *Ornithoctonus huwena*[83]. These venomous toxins have diversified their amino acid sequence causing a positive net charge on the all-atom Kunitz landscape (see Figure 2A). Reports have shown that toxins possessing a positive surface are most likely to target ion channels [84].

To date, only a few salivary secreted tick Kunitz peptides have been structurally resolved; however, these few reports reveal the venomous nature of these salivary peptides compared with other Kunitz structures from venomous animals. Figure 2A shows that the archetypal Kunitz fold is highly conserved for these tick salivary peptides and that they are structurally similar to HWTX-XI and LmKKT-1a. These structurally resolved tick salivary peptides show a structural conservation in their disulfide bridges (indicated by roman numerals), β-hairpin and the C-terminus α-helix. The only deviant from the archetypical Kunitz tertiary structure is Ra-KLP, since it is missing the second (II) disulfide bridge and possess a modified apex due to two atypical disulfide bridges (1 and 2; Figure 2A). Figure 2A also shows that the electrostatic potential of HWTX-XI and LmKKT-1a is strikingly similar to both TdPI and Ra-KLP, both from the salivary glands of *R. appendiculatus*. Ra-KLP has been reported as an ion channel modulator [85] like LmKKT-1a and HWTX-XI with no protease activity. It remains to be tested, however, if and how TdPI affects ion channels. Figure 2B shows a Cα backbone protein structural alignment of the represented Kunitz peptides. The root mean square deviation compared with HWTX-XI does not exceed 3 Å (TAP = 3 Å; ornithodorin = 2.4 Å; boophilin = 1.7 Å; TdPI = 2.8 Å; Ra-KLP = 2.8 Å); the structural difference with LmKKT-1a slightly varies from these deviations, but does not exceed 3.3 Å. Regardless of the conservative nature in the Kunitz fold, these tick salivary peptides display functional versatility and target different short-term physiological processes [85-89]. Therefore, as one of the most abundant tick salivary protein families [80], we consider Kunitz peptides as a typical example of a venomous agent that fit all five properties (i-v) referred above and described by Fry and colleagues [2].

### Cystatins

**Figure 3:**
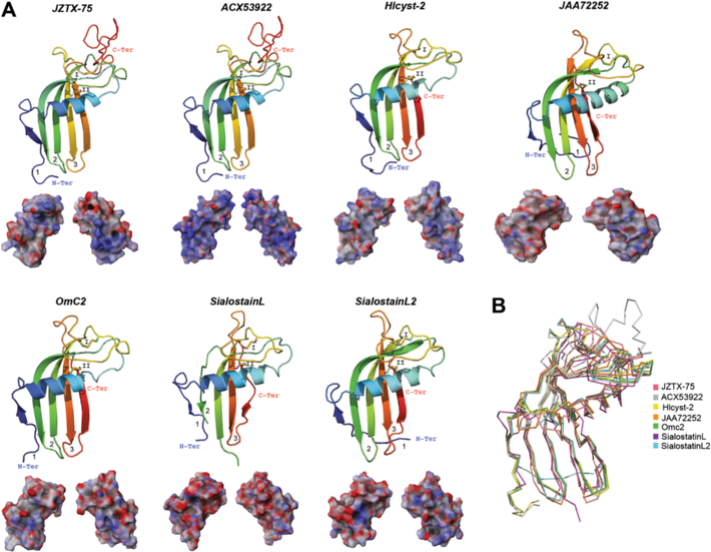
**Tertiary structures of tick salivary cystatins.** Panel **A** displays predicted tertiary structure of a cystatin from spider venom (JZTX75; GenBank: ABY71743) and, from tick salivary glands, we present three predicted cystatin structures (GenBank: ACX53922, JAA72252 and Hlcyst2-ABV71390) and three crystal structures (PDBs: OmC2-3L0R; sialostatinL-3LI7; sialostatinL2-3LH4). The tertiary structures depict the conserved disulfide bridges (indicated by roman numerals), β-sheets, the α-helix, and the inhibitory loop regions (1-3). All structures are colored from the N-terminus (blue) to the C-terminus (red). Below each tertiary structure is the respective electrostatic potential in 180° turns (blue = positive; red = negative; white = neutral). A tertiary structural alignment in Panel **B** depicts the Cα protein backbone (color codes for each structure is presented on the right). (Note: For Panel **A** we used the C-terminus domain for sialostatinL).

Although cystatins have been identified from the venomous glands of spiders [90], snakes [91] and caterpillars [92], the venomous function of these cystatins remain elusive. Protease inhibition is the most common activity reported for these cystatins, as in one of the earliest studied cystatins isolated from the venom glands of the African puff adder (*Bitis arietans*) that inhibits papain, cathepsin B and dipeptidyl peptidase I [93]. The inhibitory sites of cystatins that bind during protein-protein interactions are the N-terminal loop and the two β-hairpin loop regions (indicated in Figure 3A as 1-3). A total of 95 cystine knot toxins have been identified in the venom glands of the tarantula *Chilobrachys jingzhao* and several of these toxins were reported to inhibit ion channels [90]. Two disulfide bonds form cystine knot toxins with their backbone connected by a third disulfide bond and the overall structure is invariably stabilized by β-sheets. Examples of these cystine knot toxins are Kunitz and defensin peptides. Although its toxic effects remain elusive, the cystatin JZTX-75 was among the 95 cystine knot toxins identified in the venom glands of the tarantula *C. jingzhao*[90]. The predicted tertiary structure of JZTX-75 (shown in Figure 3A) possesses a slightly positive electrostatic potential.

Over 80 cystatins have been reported in the salivary glands of hard and soft tick species [57,61,63,64,77,94]. For a full description on the physiological role of tick cystatins refer to [67]. In general, tick cystatins are potent inhibitors of papain-like cysteine proteases and play important roles during tick feeding. Tick salivary cystatins have been shown to serve as host immune modulators but their basic functions in tick saliva are unknown. A secreted cystatin has also been identified in the tick gut of *H. longicornis* that increases in expression during feeding on its host (Hlcyst-2; Figure 3A) [95]. Three crystal structures of cystatins secreted by tick salivary glands of *I. scapularis* (sialostatinL and sialostatinL2) and *O. moubata* (OmC2) have been resolved. Although the binding of these tick cystatins remain elusive, an *in silico* study showed that these inhibitory loop regions for sialostatinL2 are conserved (Figure 3) [67]. A recent study showed that several tick cystatins were constantly expressed during a 5-day feeding period; among these was the cystatin ACX53922 [96]. Compared with the other five cystatins in Figure 3A, ACX53922 displays a more positive electrostatic potential throughout its all-atom landscape while still maintaining the archetypal tertiary backbone structure (Figure 3B; all structures have <3.0 Å root mean square deviation compared with JZTX-75).

### Defensins

**Figure 4:**
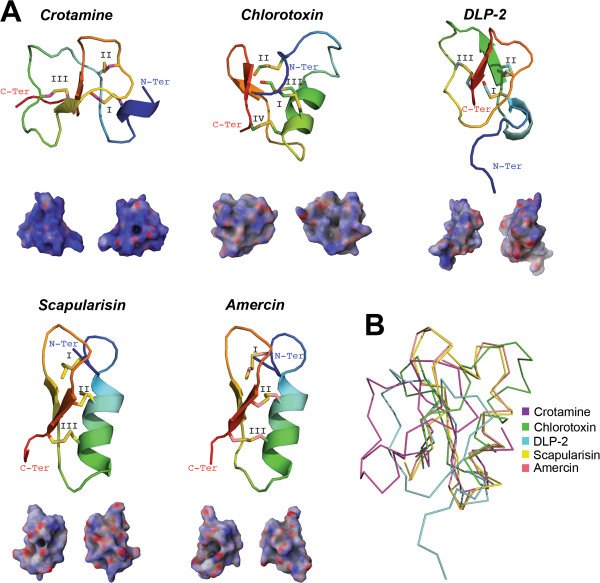
**Tertiary structures of tick salivary defensin peptides.** Panel **A** displays the crystal structure of toxins from rattlesnake (crotamine; PDB: 1Z99), scorpion (chlorotoxin; PDB: 1CHL) and platypus (DLP-2; PDB: 1D6B), and two predicted tertiary structures from tick salivary glands (GenBank: scapularisin-AAV74387 and amercin-ABI74752). The tertiary structures depict the conserved disulfide bridges (indicated by roman numerals), loops, β-sheets that forms the β-hairpin, and the α-helix. All structures are colored from the N-terminus (blue) to the C-terminus (red). Below each tertiary structure is the respective electrostatic potential in 180° turns (blue = positive; red = negative; white = neutral). A tertiary structural alignment in Panel **B** depicts the Cα protein backbone (color codes for each structure is presented on the right).

As in the Kunitz family, defensin peptides are widely distributed among the kingdoms of life as they are found in plants [97], jellyfish, sponges, nematodes, crustaceans, arachnids, insects, bivalves, snails, sea urchins, birds [98] and mammals [99], including humans [100]. The two structural classes of defensins are, (i) those exclusive to vertebrate known as α- β- and θ-defensins [101] and (ii), the most extended, possessing a simple structural motif known as the cysteine-stabilized α-helix and β-sheet (CSαβ) [102] as those depicted in Figure 4A. Defensins have a wide range of biological functions, varying from sweet-tasting proteins to antimicrobial peptides (AMP) [102]. Recruitment of defensins has been reported in scorpion [103,104], snake [105], lizard [106], platypus [107] and spider [108] venom glands. The main function of defensins as animal toxins is to target ion channels [102]. Defensin molecules can also possess multiple biological functions that include ion channel modulation, antimicrobial and antifungal activity, such as crotamine, the β-defensin myotoxin from the rattlesnake *Crotalus durissus terrificus*[109]. In contrast, although isolated from the spider venom of *Ornithoctonus hainana*, the Oh-defensin was shown so far to only possess antimicrobial activity [108].

A single experimentally induced genetic deletion or mutation transforms a non-toxic defensin into a neurotoxin [99], thus, reinforcing the concept that toxic molecules are recruited from ancestral proteins possessing a non-toxic physiological function [2]. This also suggests the evolutionary steps necessary for recruiting defensins in the venom of venomous animals [99]. In agreement with the functional diversity of defensins, it is evident, from reported crystal structures, that the tertiary structure is highly divergent (Figure 4B; all have ~3.5 Å root mean square deviation compared with crotamine). Defensins in ticks show a diverse expression pattern, thus they have been isolated from tick haemocytes, gut, intestine, ovaries, malpighian tubules and fat body [110,111]. Nevertheless, some of these defensins are exclusively expressed in tick salivary glands [112]. The most widely reported defensin structure in both hard and soft tick species is the CSαβ [113]. The only function assigned to the majority of characterized tick defensins, thus far, is AMP [65,110-112]; however, haemolytic activity was also recently reported for *I. ricinus* and *H. longicornis* defensins [111,114]. This obviously does not exclude the possibility that tick defensins may have other toxic functions in the vertebrate host. Other types of cysteine-rich AMPs from ticks were found to inhibit serine proteases [115], specifically chymotrypsin and elastase [116]. Furthermore, some tick defensins have secondary and tertiary structures similar to membrane potential modulators, such as scorpion neurotoxins, snake safaratoxins and plant γ-thionins [117] suggesting a toxic role for these tick defensins. Another example of evolutionary convergence between ticks defensins and toxic defensins are the recently discovered multigenic defensin-like peptides, scasin and scapularisin, from the toxicoses-inducing tick *I. scapularis* (scapularisin is shown in Figure 4A) [65]. Of these functionally uncharacterized novel defensin-like peptides, scasins show a strong positive selection acting on the whole molecule [65], an evolutionary pattern observed before in conotoxins from the molluscs of the genera Conus [118] that act as ion channel modulators [119]. Nevertheless, further studies should clarify whether scapularisin and scasins are ion channel effectors, or not. As Figure 4A shows, however, tick defensins have similar electrostatic potentials as those found in snake and scorpion venom.

### Lipocalins

Lipocalins are multifunctional proteins with a β-barrel structure that share three conserved domains in their primary structure, namely, motifs 1–3. Lipocalins have been implicated in development, regeneration, and pathological processes, but their specific roles are not known [120]. In reptiles and other venomous taxa, venom systems are enriched through gene duplication [2,121], thereby increasing its functional divergence to develop a new function or neofunctionalization [47]. Neofunctionalization in tick lipocalins is a good example of functional diversification found in the venom of several venomous taxa [2].

**Figure 5:**
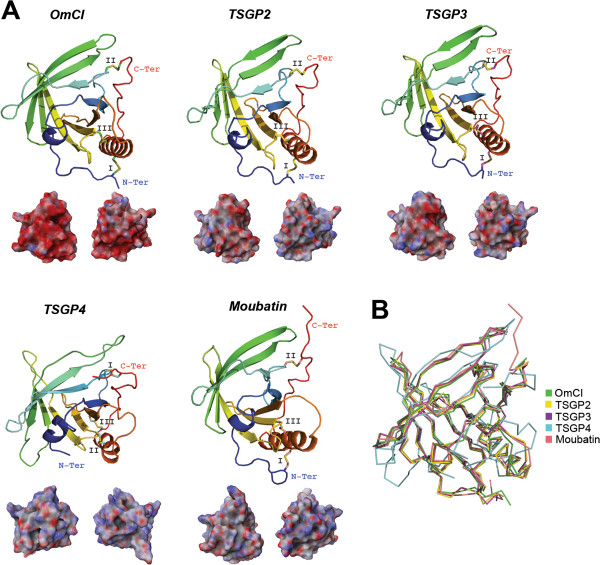
**Tertiary structures of tick salivary lipocalins.** Panel **A** displays the crystal structure of the tick lipocalin OmCI from *O. moubata* (; PDB: 3ZUO) and four predicted tertiary structures from the tick toxins, namely TSGP2, TSGP3, TSGP4 from *O. savignyi* and moubatin, also from *O. moubata* (respective UniProt: Q8I9U1, Q8I9U0, Q8I9T9 and Q04669). The tertiary structures depict the conserved disulfide bridges (indicated by roman numerals), loops, β-sheets that forms the β-hairpin, and the α-helix. All structures are colored from the N-terminus (blue) to the C-terminus (red). Below each tertiary structure is the respective electrostatic potential in 180° turns (blue = positive; red = negative; white = neutral). A tertiary structural alignment in Panel **B** depicts the Cα protein backbone (color codes for each structure is presented on the right). All structures have <2.2 Å root mean square deviation compared with OmCI.

Lipocalin-scaffolds have frequently been recruited as tick salivary components. Examples of toxin recruitment in tick salivary glands are the sand tampan toxins (TSGP) from *O. savignyi*, an abundant protein group that form a phylogenetic cluster with members of the tick lipocalin protein family, suggesting that they originated via gene duplication [21,69-71]. Three TSGPs were isolated from salivary gland extract in the tampan *O. savignyi*: the toxins TSGP2 that produces ventricular tachycardia, TSGP4 that produces Mobitz-type ventricular block, and the non-toxic TSGP3 that inhibits platelet aggregation [21,69,71]. Two other lipocalins closely related to TSGP2/TSGP3 are moubatin (platelet aggregation inhibitor; [122]) and OmCI from the soft tick *O. moubata* (complement inhibitor of C5 activation) [123]. It is worth mentioning that OmCI [124], TSGP2 and moubatin have dual action and triple action was reported for TSGP3 [71]. These tick salivary lipocalins are depicted in Figure 5. Multifunctionalization is a common trait found in the toxins of *Lonomia obliqua*[125] and, as previously mentioned, for defensin peptides found in some venomous systems.

Due to a recent European upsurge of allergic reactions caused by the pigeon tick *A. reflexus* (e.g. [126]) a major allergen was identified (Arg r1) that is homologous to toxic lipocalins from *O. savignyi*[127,128]. Histamine-binding proteins were also described as lipocalins from the tick saliva of *R. appendiculatus*[46] and recently, a novel group of lipocalins was reported from metastriate tick saliva possessing a modulatory activity on dendritic cells [49]. Another lipocalin was isolated from *A. monolakensis* (AM-33) that binds to cysteinyl leokotrienes with high affinity, avoiding endothelial permeability and formation of edema, thus ensuring the tick to replete an erythrocyte-rich meal [129].

### Phospholipase A2

The phospholipase A_2_ (PLA_2_) superfamily are ubiquitously found throughout the animal kingdom to catalyze the hydrolysis of ester bonds in a variety of different phospholipids producing lysophospholipids and free fatty acids that play important physiological roles [130]. The PLA_2_ superfamily includes five distinct enzyme types that are composed of 15 groups with many subgroups depending if they are secreted, cytosolic, calcium-independent or based on their specific target [131]. The PLA_2_ superfamily has also been recruited via convergent evolution into the toxic arsenal of cephalopods, cnidarians, insects and arachnids [2]. In the venom of reptiles, PLA_2_ appears as an antiplatelet aggregation factor [132], a myotoxin and a neurotoxin [133].

**Figure 6:**
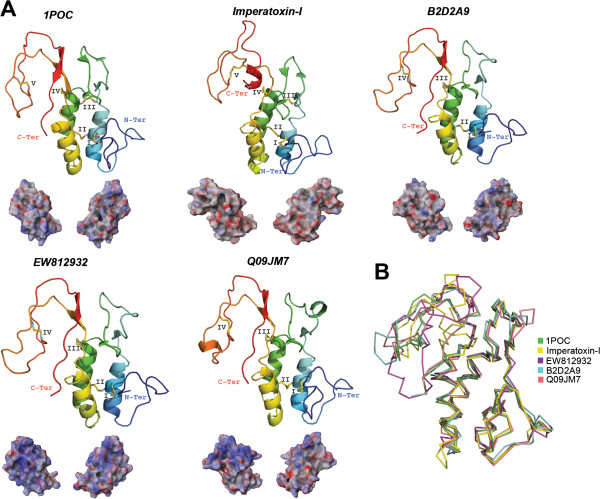
**Tertiary structures of tick salivary PLA**_**2**_**.** Panel **A** displays the crystal structure of the PLA_2_ from bee venom of *A. mellifera* (PDB: 1POC) and four predicted tertiary structures, three tick PLA_2_ (respective UniProt: B2D2A9, Q09JM7 and the translated sequences GenBank: EW812932) and the scorpion toxin, imperatoxin-I (UniProt: P59888). The tertiary structures depict the conserved disulfide bridges (indicated by roman numerals), loops, and the α-helix. All structures are colored from the N-terminus (blue) to the C-terminus (red). Below each tertiary structure is the respective electrostatic potential in 180° turns (blue = positive; red = negative; white = neutral). A tertiary structural alignment in Panel **B** depicts the Cα protein backbone (color codes for each structure is presented on the right).

As previously stated, tick toxicoses is related to feeding and feeding cycle. Tick salivary gland PLA_2_ activity was found to be higher in engorged *A. americanum* compared with unfed ticks and this increase was correlated with salivary gland secretion [134]. Although the function remains unknown, the PLA_2_ activity found in the saliva of *A. americanum* was suggested to play an important role during prolonged tick feeding (10-14 days for *A. americanum*) [73]. The salivary PLA_2_ from *A. americanum* is alkaline (pH: 9.5), as previously reported for PLA_2_ from bee and snake venom [73], and does not contribute to the anticoagulant activities found in the saliva of *A. americanum*[135], but possess hemolytic activity [73]. The PLA_2_ from both tick [136] and rattlesnake [137] possess antibacterial activities, suggesting a functional confluence between these two venomous species. Nevertheless, the PLA_2_ from *A. americanum* was not inhibited by aristolochic acid [73] as previously reported for the PLA_2_ from the venomous snake, *Vipera ammodytes meridionalis*[138]. Sousa and colleagues [139] demonstrated the complexity of the toxic effects induced by venomous molecules since the PLA_2_-melitin complex in *Apis mellifera* venom acted as a vasoconstrictor on rat aorta; however, no effect was evidenced for the PLA_2_ and melitin fractions individually. New methods for testing the molecular functions of tick molecules may contribute to unravel the intricate putative toxic effects of tick salivary PLA_2_. For example, a new method for dsRNA delivery was reported using *I. scapularis* eggs and nymphs, incorporating electroporation instead of microinjection. One of the genes that were successfully silenced was a putative PLA_2_ from *I. scapularis*[58]. We compared this putative PLA_2_ from *I. scapularis* (GenBank: EW812932), and a few other putative PLA_2_ from ticks that induce toxicoses (most are depicted in Figure 1) with the crystal structure of the PLA_2_ present in *A. mellifera* venom (PDB: 1POC; Figure 6A) and a predicted model from the scorpion toxin, imperatoxin-I that targets the sarcoplasmic reticulum calcium-release channel of skeletal and cardiac muscles [140]. Although these tick PLA_2_ have <1.8 Å root mean square deviation compared with *A. mellifera* (Figure 6B), they have lost the disulfide bridge IV that is present in *A. mellifera* PLA_2_ (Figure 6A). A recent study reports that, due evolutive pressures caused during the arms race with the host(s), ticks express non-canonical variants of highly conserved protein families and these variants possess an altered disulfide bridge pattern that provide functional flexibility – e.g., Kunitz protein family from *I. ricinus*[80].

### Lectins

**Figure 7:**
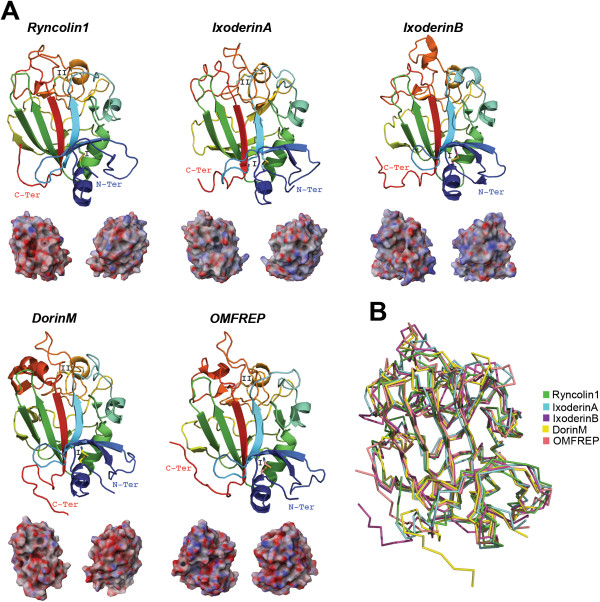
**Tertiary structures of tick salivary lectins.** Panel **A** displays five predicted tertiary structures ryncolin1 from snake venom (UniProt: D8VNS7) and four tick lectins, IxoderinA, IxoderinB, DorinM, OMFREP (respective UniProt: I6LAP5, Q5IUW6, Q7YXM0 and Q8MUC2). The tertiary structures depict the conserved disulfide bridges (indicated by roman numerals), loops, β-sheets that forms the β-hairpin, and the α-helix. All structures are colored from the N-terminus (blue) to the C-terminus (red). Below each tertiary structure is the respective electrostatic potential in 180° turns (blue = positive; red = negative; white = neutral). A tertiary structural alignment in Panel **B** depicts the Cα protein backbone (color codes for each structure is presented on the right).

Lectins can be defined as a wide variety of carbohydrate-binding proteins and glycoproteins from viruses, bacteria, fungi, protista, plants, and animals [141]. Lectins found in snake venom mainly affect blood coagulation pathways [142] and can also act as anti-angiogenic compounds [143]. In caterpillars, lectins are known to function as anticoagulants as in the *Lonomia* venom [92], but may also possess a myotoxic effect as in stonefish venom [144]. To date, tick lectin research has mainly focused on its roles in tick innate immunity (for revision see [145,146]). Earlier studies, however, showed that *R. microplus* saliva possesses lectin activity and induces immunosuppression in mice [147]. Additionally, Rego and colleagues [66,148] reported four tick lectins, two from *O. moubata* (Dorin M and OMFREP) and two from *I. ricinus* (Ixoderin A and Ixoderin B). Based on phylogenetic analysis and expression pattern, a putative role in tick innate immunity was assigned to Ixoderin A and OMFREP. The role of Ixoderin B still remains unknown since it was exclusively expressed in the salivary glands and presented sequence divergence compared with Ixoderin A and OMFREP. Figure 7A depicts these tick lectins compared with ryncolin1 from the venom of the dog-faced water snake, *Cerberus rynchops* (all structures have <1.8 Å root mean square deviation; Figure 7B), which was suggested to induce platelet aggregation and/or initiate complement activation [149]. We note that IxoderinB also (as in Kunitz and PLA_2_) does not possess the archetypal disulfide bridge pattern (II; Figure 7A). In addition to its sequence divergence to Ixoderin A and OMFREP, the missing disulfide bridge of IxoderinB may cause a more flexible fold thus diversifying its target(s).

### A comparison of salivary proteins from humans and ticks

**Figure 8:**
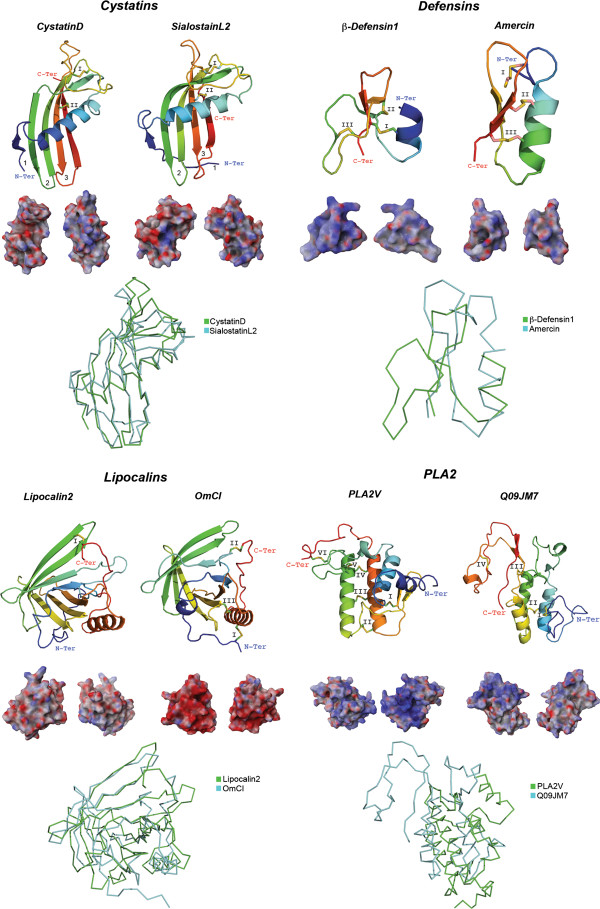
**Human and tick salivary proteins.** The panel shows tick salivary proteins described in the previous figures compared with the crystal structures of human cystainD (PDB: 1ROA), β-defensin1 (PDB: 1IJU), and lipocalin2 (PDB: 3BX8). The human salivary PLA2, GroupV (PLA2V; GenBank: AAH36792) was modeled. The tertiary structures depict the conserved disulfide bridges (indicated by roman numerals), loops, β-sheets that forms the β-hairpin, and the α-helices. All structures are colored from the N-terminus (blue) to the C-terminus (red). Below each tertiary structure is the respective electrostatic potential in 180° turns (blue = positive; red = negative; white = neutral) and tertiary structural alignment of the Cα protein backbone (color codes for each structure is presented on the right).

In order to compare the saliva of ticks to the saliva of a non-venomous animal we established a comparison between human and tick salivary systems. Recent proteomic studies have identified a total of 2698 proteins in human saliva (revised in [150]). The major protein components of human saliva are amylase, carbonic anhydrase, mucins, cystatins, proline-rich proteins, histatins, statherins and antibodies (revised in [150]), but it also contains defensins, lactoperoxidases, lysozymes and lactoferrins [151]. The complexity of the saliva from non-venomous animals (e.g., humans) is akin to that of ticks, but there are major differences in the molecular functions, the structure and the electrostatic potential of common salivary protein families. The two salivary systems present similar components such as lysozymes, cystatins, lipocalins, defensins and PLA2s. There are also differences, for example, human saliva possesses histatins and statherins, but these proteins have not been found in tick saliva. Despite that human salivary glands and the Kunitz protein family have been scientifically investigated for some time, the authors are unaware of any reports indicating the presence of Kunitz peptides (i.e., the archetypal 60 aa long peptide) in human saliva. The main reports for human Kunitz (also found in saliva) are of domains from larger proteins, e.g., immunoglobulins. Additionally, although the lectin intelectin-1 (UniProt: Q8WWA0) has been found in human saliva, its specific tertiary structure may drastically differ from those currently reported since a PSI-BLAST against the PDB was unable to retrieve a homologous structure and thus we were unable to model this human salivary lectin. In addition, human saliva does not present any allergen-like molecules; as are found in tick saliva. These differences are not surprising if we consider the different alimentary regimes these two species have and the molecular functions these two salivary systems need to perform. The phylogenetic distance between human and ticks maybe the most rational explanation for such differences. The salivary composition of the venomous mammal *D. rotundus*, however, is similar to ticks. *D. rotundus* salivary glands possess both Kunitz proteins and allergen-like molecules, while also possessing humans salivary components, like statherins and lysozymes [44]. As discussed above, this suggests that venomous animals recruit in their salivary glands a special set of proteins with specific venomous functions.For the protein families we report here, the available tertiary structures for humans (and those we were able to model) are cystatins, defensins, lipocalins and PLA2 (Figure 8). There seems to be a bimodal diversity among these salivary protein families. Figure 8 shows that cystatins and lipocalins are structurally conserved, however, the electrostatic potential differs considerably – as dramatically displayed in OmCI. The opposite is seen for defensins and PLA2s – similar electrostatic potential surface with dissimilar tertiary structures. This bimodal diversity is also evidenced within these protein families from venomous organisms compared to their human counterparts. The defensin crotamine, for instance (Figure 4), is structurally similar to human β-defensin1 (Figure 8), but their electrostatic potential and landscape differs (crotamine being more basic). It is worth mentioning that the tick salivary proteins depicted in Figure 8 are from some of the toxicoses-inducing ticks represented in Figure 1.

### Considerations on the most recent common ancestor of Parasitiformes and Pseudoscorpiones

Given the evidence provided in the previous sections, we find it necessary to reconsider ticks as venomous ectoparasites due its salivary properties and its evolutionary implications. Arthropods represent a major component of the biodiversity of life on Earth and venom systems, as an evolutionary adaptation, appear many times in Arthropoda (e.g., spiders, scorpions, wasp, bees and flies). With a few exceptions in Hexapoda (i.e., bees and some wasps), the main function of venomous molecular systems amongst Arthropods is to assist in predation [47]. Ticks are haematophagous Arachnids and, based on morphological characteristics, it has been argued that Holothyrida is the sister taxa of Ixodida [18]. This evolutionary relationship with Holotryrida, free-living mites that mainly feed on the body fluids of dead arthropods [152], has lead to inferences regarding the feeding habits of the most recent common ancestor (MRCA) of ticks, which has been described as a saprophytic organism [153] or a scavenger in which haematophagy evolved subsequently [81]. Entomophagy was another type of predatory behavior proposed for the MRCA of ticks and the cannibalism observed in some soft tick species was an argument in favor of this hypothesis [81].

**Figure 9:**
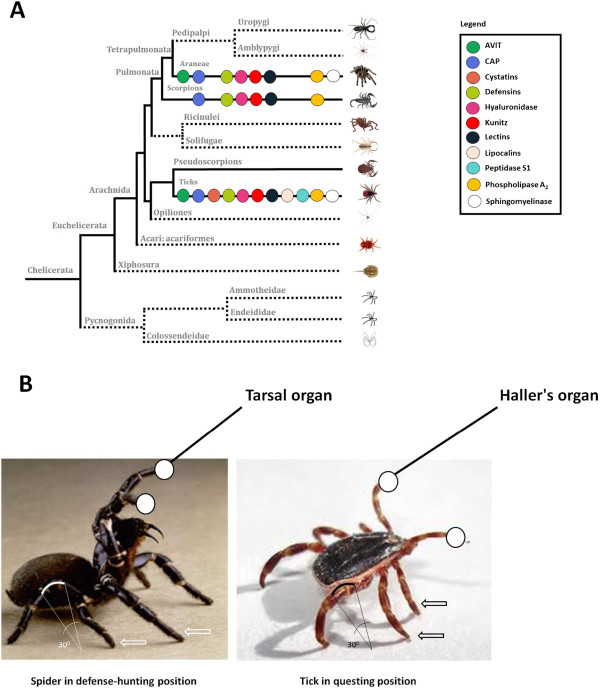
**Similarities among venomous animals in chelicerata.** Panel **A** shows the phylogenetic tree compiled from published sources [9]. Data regarding protein families in ticks, spiders and scorpions was collected from [2], as well as protein families characterized in recent studies. Panel **B** shows a spider’s defense/hunting and a tick’s questing positions (photos are not in real dimensional scale). The images depict similarities in attitude, angle of flexion in posterior legs (30˚), middle legs (arrows), and first pair of legs exposing the respective sensory organs (white circles).

Haematophagy has evolved several times from predation in Insecta; for example, triatomine and cimicid bugs evolved from predatory heteropterans and Symphoromyia (and other hemathophagous flies) evolved from predatory ones [154]. The characteristics of the MRCA of ticks are paramount in understanding the evolution of tick salivary constituents. Recently, by using maximum likelihood, Bayesian and parsimony analyses of over 41 kilobases of DNA sequence from 62 single-copy nuclear protein-coding genes, Regier and colleagues [9] presented a strong, revised and actualized phylogeny of Arthropoda. In these phylogenetic analyses, Parasitiformes (Acari) appears as a sister group of Pseudoscorpions (Figure 9A). The phylogenetic position of Pseudoscorpions in Chelicerata has caused large debate in recent years. By using 2907 amino acids from 13 different proteins, another series of phylogenetic analyses by Ovchinnikov and Masta [155] also showed that Pseudoscorpions, although not a sister group, are closely related to Parasitiformes. From both studies [9,155], we could certainly infer that Pseudoscorpions and ticks share a recent common ancestor.

Pseudoscorpions are venomous members of Chelicerata [156] that, together with spiders and scorpions [2], constitute well-known examples of venomous animals amongst Chelicerata. Our working hypothesis is that the MRCA of ticks was a venomous predator of smaller preys that later evolved to feed on larger vertebrates. In agreement with this, the feeding plasticity (as a capacity of feeding on several hosts) of the tick ancestor was recently suggested [157]. The convergence of several protein families between tick saliva and the venom of spiders and scorpions is shown in Figure 9A. This suggests a common origin in the venom systems of these three taxa (pseudoscorpions were not included in the comparison due to a lack of information regarding the venom composition of this species). Additionally, ticks are questing animals that will move actively in order to find their hosts [158-160]. Questing behavior constitute an important trait of tick ecology [158]. The evolution of questing behavior is closely related to the evolution of sensory organs like the Haller’s organ [161]. We found striking similarities in the questing behavior and position of sensory organs of ticks compared to the hunting behavior and position of sensory organs [162] of venomous spiders (Figure 9B).

## Conclusion

The literature has split the saliva of haematophagous animals into those who consider the saliva as venomous and those that consider it as a special kind of saliva or a complex cocktail of bioactive components. Just last year (2013) the *Journal of Proteomics* published two investigations on the salivary gland components of the haematophagous bat *Desmodus rotundus*, where one refers to it as venomous [44] and the other simply as salivary components [45]. In tick research, the literature predominantly considers tick saliva as a complex cocktail of bioactive components and the toxicoses induced by ticks are mainly discussed in the context of paralysis. These claims narrow the ecological implications of the venomous relationship between ticks and their hosts. To classify ticks as mere ectoparasites is limited and underestimates the venomous structure of multigenic protein families in tick saliva. Therefore, we propose to consider ticks as venomous ectoparasites based on the intrinsic properties of tick saliva.

## Methods

### Tertiary protein modeling, structural alignment and electrostatic potential

Predicted tertiary models were generated using the Phyre2 server [163]. All predicted models were then refined via minimization and hydrogen-bond network was optimized by means of side chain sampling using the Schrodinger’s Maestro Protein Preparation Wizard [164]. The structural deviations (root mean square deviation) were calculated using the protein structural alignment tool, from the Maestro software. Electrostatic potentials were calculated using the Poisson-Boltzmann equation also implemented in the Maestro software.

## Abbreviations

AMP: Antimicrobial peptides; CSαβ: Cysteine-stabilized α-helix and β-sheet; Ig: Immunoglobulin; MRCA: Most recent common ancestor; PLA_2_: Phospholipase A_2_; PDB: Protein databank; Th2: T helper type 2.

## Competing interests

Both authors declare that they have no competing interests.

## Authors’ contributions

Both authors have contributed equally. Both authors read and approved the final manuscript.
